# Blood–Brain Barrier Disruption Is Not Associated With Disease Aggressiveness in Amyotrophic Lateral Sclerosis

**DOI:** 10.3389/fnins.2021.656456

**Published:** 2021-10-29

**Authors:** Tino Prell, Benjamin Vlad, Nayana Gaur, Beatrice Stubendorff, Julian Grosskreutz

**Affiliations:** ^1^Department of Geriatrics, Halle University Hospital, Jena, Germany; ^2^Department of Neurology, Jena University Hospital, Jena, Germany; ^3^Precision Neurology, University of Lüebeck, Lüebeck, Germany

**Keywords:** neurodegeneration, blood-brain barrier, inflammation, amyotrophic lateral sclerosis (ALS), disease aggressiveness

## Abstract

The pathogenesis of the fatal neurodegenerative condition amyotrophic lateral sclerosis (ALS) remains to be fully understood. Blood–brain barrier damage (BBBD) has been implicated as an exacerbating factor in several neurodegenerative conditions, including ALS. Therefore, this cross-sectional study used the novel D50 progression model to assess the clinical relevance of BBBD within a cohort of individuals with either ALS (*n* = 160) or ALS mimicking conditions (*n* = 31). Routine laboratory parameters in cerebrospinal fluid (CSF) and blood were measured, and the ratio of CSF to serum albumin levels (Qalb) was used as a proxy measure of BBBD. In the univariate analyses, Qalb levels correlated weakly with disease aggressiveness (as indicated by individual D50 values) and physical function (as measured by ALS Functional Rating Scale). However, after adjustment for cofactors in the elastic net regularization, only having limb-onset disease was associated with BBBD. The results reported here emphasize the clinical heterogeneity of ALS and the need for additional longitudinal and multi-modal studies to fully clarify the extent and effect of BBBD in ALS.

## Introduction

Amyotrophic lateral sclerosis (ALS) is a fatal neurodegenerative disease that predominantly affects motor neurons of both the brain and the spinal cord. This leads to muscular atrophy and paralysis, with the majority of patients succumbing to respiratory failure 3–4 years from symptom onset. Blood–brain barrier (BBB) damage (BBBD) has been reported in several neurodegenerative conditions (Sweeney et al., 2018). The BBB plays a crucial role in maintaining the internal milieu of the central nervous system (CNS) and the function of neuronal cells. It is a continuous endothelial membrane consisting of non-fenestrated vessels that regulates the transfer of cells, ions, and molecules between the CNS and the blood. Damage to the BBB results in the influx of neurotoxic blood-derived cellular debris and potentially also microbial pathogens into the brain, thus triggering inflammatory responses and associated neurodegeneration. Prior post-mortem studies have reported the leakage of blood-derived proteins and pericyte and endothelial degeneration in ALS patients (Henkel et al., 2009; Miyazaki et al., 2011; Garbuzova-Davis et al., 2012; Kwan et al., 2012; Winkler et al., 2013). Structural and functional BBBD were demonstrated in an animal model of ALS at early stage disease and worsened with disease progression (Garbuzova-Davis et al., 2007). Analysis of albumin and other serum-derived proteins in cerebrospinal fluid (CSF) and measurements of albumin CSF/serum quotient (Qalb) have indicated both a compromised BBB in living ALS patients and an increased Qalb in 40% of individuals (Brettschneider et al., 2006; Winkler et al., 2013). Despite these findings, the clinical implications of BBBD in ALS remain to be fully understood. Therefore, the present study aimed to address if higher disease aggressiveness in ALS is associated with BBBD.

## Methods

### Participants and Assessments

In this observational study, 200 subjects with either ALS or ALS mimicking conditions (mimics) were consecutively recruited between January 2015 and January 2018 from the Department of Neurology at Jena University Hospital. Nine patients were excluded due to missing data. Written informed consent was obtained from all participants, and the study was approved by the local ethics committee of Jena University Hospital (#3633-11/12). The study was performed in accordance with the Declaration of Helsinki and its later amendments or comparable ethical standards.

Diagnosis of ALS was based on the Awaji criteria, and ALS patients had definite or probable ALS as determined by the revised El-Escorial criteria (Brooks et al., 2000; de Carvalho et al., 2008). Reasons for admission to hospital included diagnostic workups to either confirm or rule out an ALS diagnosis. ALS mimics had muscle weakness and wasting for at least 6 months in at least one body region without fulfilling the Awaji criteria or revised El-Escorial criteria. Assessments included age, gender, onset type (bulbar or limb), disease duration since first motor symptom in months, and the ALS Functional Rating Scale Revised (ALSFRS-R) to quantify physical impairment (Cedarbaum et al., 1999). The ALSFRS-R rates 12 daily activities from 0 to 4, where 0 equals no function at all, and 4 equals normal function. The total score thus ranges from 0 to 48.

In addition, we obtained blood tests (complete blood count, albumin, biochemistry, and C-reactive protein) to rule out systemic inflammation and performed lumbar puncture to collect CSF. CSF samples were obtained by lumbar puncture with aseptic technique at the L3–L4 or L4–L5 intervertebral spinous process space, using a 22- or 21-gauge needle. Standard chemical parameters, including cell count, albumin, glucose, and total protein concentration, were assessed. Albumin serves as a reference protein for BBB function because it is derived exclusively from blood. The ratio of CSF to serum albumin levels (Qalb) with reference to age-specific cut-off values was used as a proxy measure of BBBD (Reiber, 1994; Petereit et al., 2007). Albumin was separated by nephelometry.

Disease aggressiveness was calculated using the D50 model (Prell et al., 2019, 2020). The D50 model describes the disease course of individuals with ALS as a sigmoidal state transition from full health (ALSFRS-R = 48) to complete functional loss (ALSFRS-R = 0). The curve is calculated by iterative fitting of ALSFRS-R scores taking into account that progression in ALS is non-linear and highly heterogeneous. Therefore, the D50 is a measure of overall disease aggressiveness and is the estimated time taken in months for the ALSFRS-R to drop to 24 points. It can therefore summatively describe individual disease aggressiveness independent of the assessment time-point.

### Statistical Analysis

The SPSS software package (version 25.0; IBM Corporation, United States) and R (R Foundation for Statistical Computing, Vienna, Austria, version 4.0.2) were used for all statistical analyses. Prior to statistical analysis, data were checked for outliers. The primary outcome measure was BBBD as indicated by the Qalb. Group comparisons were done using *t*-test, Mann–Whitney test, or the χ^2^-test or Fisher’s exact test. Spearman’s correlation was used to assess associations between clinical and biochemical variables. Elastic net regularization was applied to determine predictors (age, gender, onset type, D50, disease duration) of BBBD (Zou and Hastie, 2005). Here, variable selection is performed by shrinking parameters toward zero and attenuating overfitting, a well-known problem in common models using stepwise regression. Ten-fold cross-validation was applied to choose the best model with the lowest mean cross-validated error. Within the elastic net algorithm, variables remain in the model if the prediction error averaged over the 10 cross-validation samples is reduced. Regression coefficients of the model with odds ratios are reported. Elastic net regularization was performed with the package *glmnet* in R. For all the analyses, *p*−values < 0.05 were considered statistically significant.

Anonymized data from this study will be shared with qualified investigators on request.

## Results

The entire cohort included 160 patients with ALS and 31 patients with ALS mimic conditions; detailed demographic characteristics are presented in Table 1. BBBD was evident in 29 (18.1%) patients with ALS and in 3 (9.7%) ALS mimics. Within the ALS sub-cohort, BBBD was more frequently noted in patients with limb onset (*n* = 25, 24.3% vs. *n* = 4, 7% in bulbar onset; *p* = 0.007) (Table 2). Additionally, ALS patients with BBBD had higher total CSF protein, higher CSF albumin, and higher Qalb than patients without BBBD (Table 2). ALS patients with or without BBBD did not differ in terms of ALSFRS-R, disease duration, and D50 (Table 2). The Qalb was higher in male (*M* = 7.1 ± 2.9) than in female subjects (*M* = 6.0 ± 2.1) (*p* = 0.007), but did not differ between bulbar and limb onset (*p* = 0.36).

**TABLE 1:** Characteristics of patients with amyotrophic lateral sclerosis (ALS) and ALS mimics.

		**ALS**		**ALS mimic**		p*
		**N**	**%**	**N**	**%**	
Gender	Male	88	55.0	17	54.8	0.98
	Female	72	45.0	14	45.2	
BBBD	No	131	81.9	28	90.3	0.25
	Yes	29	18.1	3	9.7	
Onset type	Bulbar	57	35.6			
	Limb	103	64.4			

		**M**	**SD**	**M**	**SD**	

Age (years)		62.4	10.9	60.4	14.9	0.475
ALSFRS-R		36.6	7.5			
D50 (months)		35.4	24.2			
Disease duration (months)		15.7	15.4			
Total protein in CSF (mg/L)		435.7	138.0	384.8	133.2	0.061
Cell count in CSF (cells/μl)		1.6	1.7	1.6	1.5	0.907
Albumin in CSF (mg/L)		274.3	105.8	224.3	75.5	0.016
Albumin in serum (g/L)		43.1	24.1	39.4	7.2	0.412
Albumin ratio (CSF/serum) *10-3		6.7	2.6	5.7	2.2	0.069

*Comparison between ALS and ALS mimic.

**TABLE 2:** Characteristics of ALS patients with and without blood–brain barrier damage (BBBD).

		**No BBBD**		**BBBD**		**p**
		**N**	**%**	**N**	**%**	
Gender	Male	69	52.7	19	65.5	0.21*
	Female	62	47.3	10	34.5	
Onset type	Bulbar	53	40.5	4	13.8	0.007*
	Limb	78	59.5	25	86.2	

		**M**	**SD**	**M**	**SD**	

Age (years)		62.5	11.1	62.2	9.7	0.913^#^
ALSFRS-R		37.0	7.2	34.7	8.7	0.206^#^
D50 (months)		36.1	24.2	32.4	24.6	0.456^#^
Disease duration (months)		15.9	16.4	14.8	10.2	0.730^#^
Total protein in CSF (mg/L)		392.6	78.6	628.8	178.0	<0.001^#^
Cell count in CSF (cells/μl)		1.6	1.8	1.6	1.1	0.067^#^
Albumin in CSF (mg/L)		241.7	60.6	423.1	137.6	<0.001^#^
Albumin in serum (g/L)		41.4	3.8	51.2	56.3	0.373^#^
Albumin ratio (CSF/serum) *10-3		5.8	1.5	10.6	3.1	<0.001^#^

*^#^Comparison between individuals with and without blood–brain barrier damage (BBBD).*

*Comparison of BBBD presence/absence between male/female and bulbar/limb.

**FIGURE 1:**
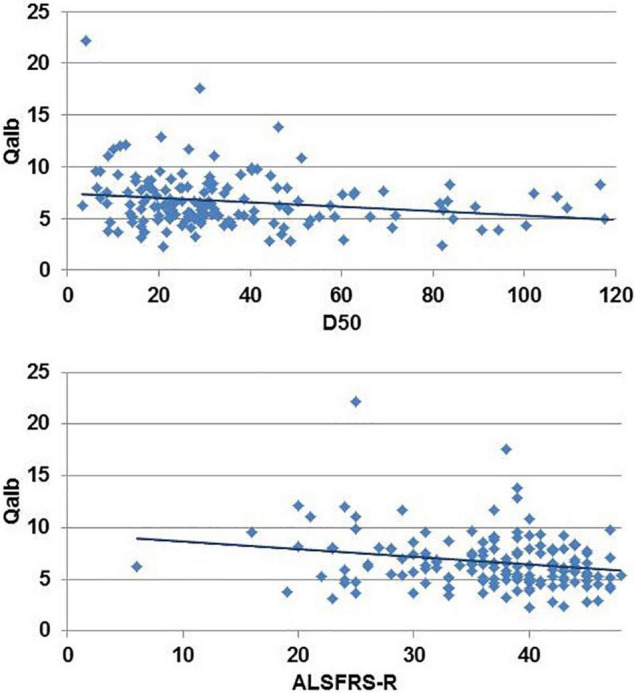
Univariate correlation between disease aggressiveness (D50) and revised ALS-Functional Rating Scale Revised (ALSFRS-R) with the albumin CSF/serum quotient (Qalb).

Univariate analyses revealed a weak correlation between Qalb and (1) D50 (*r* = −0.19, *p* = 0.013) and (2) the ALSFRS-R (*r* = −0.19, *p* = 0.015) (Figure 1); no significant correlations were noted with age or disease duration. In the elastic net model, BBBD was only associated with the onset type; having limb-onset disease was associated with a 4.2-fold increased risk for BBBD (*p* = 0.011). However, in the model, D50 was not associated with BBBD. This means, with reference to our hypothesis, that BBBD is not associated with higher disease aggressiveness in ALS.

## Discussion

BBBD can occur in several neurodegenerative diseases like Alzheimer’s disease, Parkinson’s disease, and ALS. As maintenance of BBB integrity is essential for proper neuronal and synaptic functioning, the loss of its integrity results in vascular dysfunction and a reduction in cerebral blood flow (Sweeney et al., 2018). Following disruption, the influx of albumin into the CSF is increased resulting in perivascular edema accompanied by obstruction of microcirculation. The ensuing hypoxia can favor neurodegenerative processes (Kisler et al., 2017). We hypothesized that this process could be associated with increased disease aggressiveness in ALS. While there was a weak univariate correlation between D50 and Qalb, after correction for cofactors in the elastic net model, BBBD was not associated with disease aggressiveness. Therefore, our results indicate that disease aggressiveness is not related to BBBD *per se*. We only noted an increased risk for BBBD in ALS patients with limb onset. This is surprising given that it is bulbar rather than limb-onset disease that is associated with worse prognostic outcomes (Calvo et al., 2017). Conversely, this provides further evidence for the phenotypic heterogeneity of ALS and has important implications for both biomarker and therapeutic development: different onset types should be regarded as separate entities. Given that Qalb was not related to disease duration, we further assume that BBBD is disease inherent and does not occur more frequently during the disease course. However, longitudinal CSF-based studies are needed to validate this.

This study is not without its limitations. While Qalb has been routinely used as an indicator of BBBD, there are obvious limitations associated with doing so. To begin with, albumin CSF levels can be directly influenced by both the leakage site and uptake by the glial and neuronal parenchyma in the CNS. Qalb therefore depends on the CSF turnover rate and dynamics of CSF influx. In addition, CSF albumin levels can be influenced by proteolytic cleavage and albumin uptake by brain macrophages, microglia, astrocytes, neurons, and oligodendrocytes and may therefore underestimate the degree of BBBD (Sweeney et al., 2018). Conversely, decreased CSF reabsorption could elevate Qalb, leading to false-positive results that might not reflect genuine BBBD (Sweeney et al., 2018). Moreover, it would be useful to study additional marker of BBBD (e.g., claudin) in order to understand the pathological role of BBBD in ALS. Also, the blood–spinal cord barrier (BSCB) could be of interest for future studies. The BSCB seems to be a relatively independent entity with several structural and functional differences to the BBB (Bartanusz et al., 2011). However, alterations of the BSCB in patients with ALS remain to be elucidated (Sasaki, 2015). Future studies may benefit from adopting a cross-disciplinary approach to measure BBBD with multiple modalities, including microbleed T2^∗^-weighted MRI, and measuring CSF levels of blood-derived biomarkers. Finally, the cross-sectional design of the present study does not allow causal inferences, and we further recommend longitudinal studies to examine the association between the ALS disease course and BBBD.

## Data Availability Statement

The raw data supporting the conclusions of this article will be made available by the authors, without undue reservation.

## Ethics Statement

The studies involving human participants were reviewed and approved by the Ethics Committee of the Jena University Hospital (#3633-11/12). The patients/participants provided their written informed consent to participate in this study.

## Author Contributions

TP and JG conceptualized and designed the study. TP, BV, NG, and BS assisted with data collection and analysis. TP wrote the manuscript. All authors contributed to the compilation of the manuscript and revisions for intellectual content.

## Conflict of Interest

The authors declare that the research was conducted in the absence of any commercial or financial relationships that could be construed as a potential conflict of interest.

## Publisher’s Note

All claims expressed in this article are solely those of the authors and do not necessarily represent those of their affiliated organizations, or those of the publisher, the editors and the reviewers. Any product that may be evaluated in this article, or claim that may be made by its manufacturer, is not guaranteed or endorsed by the publisher.

## Abbreviations

ALS: amyotrophic lateral sclerosis
ALSFRS-R: Amyotrophic Lateral Sclerosis Functional Rating Scale Revised
BBB: blood–brain barrier
BBBD: blood–brain barrier damage
CNS: central nervous system
CSF: cerebrospinal fluid
Qalb: albumin CSF/serum quotient.
